# Behavior Modification Techniques on Patients with Chronic Pain in the Context of COVID-19 Telerehabilitation: An Umbrella Review

**DOI:** 10.3390/ijerph19095260

**Published:** 2022-04-26

**Authors:** Ferran Cuenca-Martínez, Joaquín Calatayud, Luis Suso-Martí, Clovis Varangot-Reille, Aida Herranz-Gómez, María Blanco-Díaz, José Casaña

**Affiliations:** 1 Exercise Intervention for Health Research Group (EXINH-RG), Department of Physiotherapy, University of Valencia, 46010 Valencia, Spain; ferran.cuenca@uv.es (F.C.-M.); clovis.varangotreille@gmail.com (C.V.-R.); aidahergo10@gmail.com (A.H.-G.); jose.casana@uv.es (J.C.); 2 Surgery and Medical Surgical Specialties Department, Faculty of Medicine and Health Sciences, University of Oviedo, 33003 Oviedo, Spain; blancomaria@uniovi.es

**Keywords:** telehealth, e-health, COVID-19, pain management

## Abstract

The aim of this systematic review (SR) of SRs was to assess the effectiveness of telerehabilitation based on behavior modification techniques (t-BMT) in patients with chronic musculoskeletal pain. We searched in PubMed, PEDro, Web of Science, CINAHL, PsycINFO, and Google Scholar (January 2022). The outcome measures were pain intensity, disability, psychological distress, pain-related fear of movement, disease impact, depressive symptoms, anxiety symptoms, and physical function. This review was previously registered on the international prospective register of systematic reviews PROSPERO (CRD42021262192). Methodological quality was analyzed using the AMSTAR and ROBIS scales, and the strength of evidence was established according to the Physical Activity Guidelines Advisory Committee grading criteria. Four SRs with and without meta-analyses covering 25 trials and involving 4593 patients were included. Of the three SRs that assessed pain intensity, two reported a significant decrease compared to usual care. Contradictory results were also found in the management of psychological distress, and of depressive and anxiety symptoms. However, two reviews found that t-BMT has significant effects on disability, and one review found that t-BMT seems to be effective for improving pain-related fear of movement and disease impact. Finally, one review found that t-BMT does not seem to be an effective modality to improve physical function. The quality of evidence was limited for all outcomes assessed. The results obtained showed that t-BMT was effective in improving disability, disease impact, and pain-related fear of movement, but it was not effective in improving physical function in patients with chronic pain. Mixed evidence was found for pain intensity, psychological distress, and depressive and anxiety symptoms, with a limited quality of evidence.

## 1. Introduction

The COVID-19 pandemic has provoked a major shift in the way we treat patients with persistent musculoskeletal pain [1]. The lack of access to clinics and the health emergency undermined face-to-face treatments [2]. An online survey has shown that 80% of chronic pain patients have experienced a worsening of their symptoms during the pandemic and an increase in the interference of pain with daily life, including work and physical activities [3]. It is evident that not treating these patients will lead to severe consequences for them [1]. In this context, telerehabilitation appears as a key solution to counteract the burden of the COVID-19 pandemic on patients with persistent musculoskeletal pain [1,4]. Telerehabilitation is the use of a technology-based virtual platform (e.g., the Internet, mobile applications) to provide various aspects of therapeutic rehabilitation remotely [5]. The use of telecommunication technologies seems promising [6], especially in a society where new technologies are omnipresent. Some pain services have almost quadrupled their use [7]. Suso-Martí et al. [8] showed that there were no statistically significant differences between in-person physical therapy treatment and its delivery via telerehabilitation for various chronic conditions. Physical therapy is essential for patients with severe disabilities, and telerehabilitation should not only allow the physiotherapist to enter the patient’s home, but also allow the individual to get out of lockdown [9]. However, there is no overview of the effects of telerehabilitation on patients with persistent musculoskeletal pain.

As Turk et al. [10] reported, psychosocial and behavioral factors play a significant role in the experience, maintenance, and exacerbation of pain. Part of the problem with chronic pain patients arises from how they react to their pain [11]. Behavioral modification techniques (BMTs), such as cognitive behavioral therapy, do not necessarily require the therapist to be physically present with the patient [12]. There are several systematic review articles with or without meta-analyses that have addressed this topic, but no research article has provided a general overview of the effect of telematic BMT (t-BMT) with respect to clinical endpoints and variables of interest in patients with chronic pain [6,13,14,15].

It is therefore the main aim of this review to synthesize the evidence on the effects of t-BMT in improving pain intensity, disability, psychological distress, depressive symptoms, anxiety symptoms, physical function, pain-related fear of movement, and disease impact, compared with no intervention or in-person treatment, in patients with persistent musculoskeletal pain.

## 2. Methods

This study was conducted in accordance with the Preferred Reporting Items for Overviews of Systematic Reviews, including a harm checklist (PRIO-harms), which consists of 27 items (56 sub-items), followed by a 5-stage process flow diagram (identification, screening, eligibility, inclusion, and separation of relevant studies) [16]. The review was previously registered on the international prospective register of systematic reviews PROSPERO (CRD42021262192).

### 2.1. Review Inclusion Criteria

The inclusion criteria employed in this article were based on methodological and clinical factors such as population, intervention, control, outcomes, and study design [17].

#### 2.1.1. Population

The participants selected for the articles were patients older than 18 years diagnosed with any musculoskeletal disorder that was accompanied by chronic pain (e.g., fibromyalgia, chronic lower-back pain, migraine, chronic back pain). Chronic musculoskeletal pain was defined as diseases of the muscles and their associated ligaments and other connective tissue, and of the bones and cartilage, viewed collectively, along with pain that persists over time, under to the medical subject headings (MeSH) “Musculoskeletal diseases”, “Musculoskeletal Pain”, and “Chronic Pain”. Included systematic reviews had to explicitly state that they included patients with chronic pain lasting more than 3 months in their inclusion criteria. However, if they performed a “chronic pain” sub-analysis, we included it as well. Therefore, we excluded patients with acute musculoskeletal pain, traumatic musculoskeletal injuries, or confounding comorbidities such as cancer, as well as articles that did not specify the pain duration of the analyzed patients.

#### 2.1.2. Intervention and Control

The intervention consisted of t-BMT conducted in isolation or combined with other treatment techniques. The following are some examples of known BMTs used in telerehabilitation: cognitive behavioral therapy (CBT), which is aimed at identifying and modifying the client’s maladaptive thoughts and dysfunctional behaviors through behavioral techniques to achieve change; acceptance and commitment therapy (ACT), which is based on the patient’s acceptance of their thoughts and feelings as a necessary part of their life in order to develop new and more flexible ways of thinking; self-management including exercise, which is based on self-monitoring, self-evaluation, and goal setting, among others; exposure behavioral therapy, which is based on the repeated exposure to the feared stimulus or context; and mindfulness, which is based on a moment-to-moment awareness of one’s experience without judgment. Web-based intervention was defined as the use of an online platform, through the Internet or a mobile application, to deliver therapeutic rehabilitation at a distance, according to the MeSH “Telerehabilitation”. We excluded any telerehabilitation not aimed at changing patients’ behavior. The compared groups used the following interventions: usual care; waiting lists; books or guides provided physically or remotely (by mail or e-mail) that are not specifically aimed at changing behavior; equivalent non-telerehabilitation intervention; magazine subscription control intervention; or in-person treatment.

#### 2.1.3. Outcomes

The outcomes employed to assess the effects of t-BMT were pain intensity, disability (lack or limitation of any physical or psychological faculty that makes it impossible or difficult for a person to carry out a normal activity), psychological distress, depressive symptoms, anxiety symptoms, physical function, pain-related fear of movement, and disease impact.

#### 2.1.4. Study Design

We selected systematic reviews (with or without a meta-analysis) of randomized controlled clinical trials (RCCTs) or controlled clinical trials (CCTs), and excluded systematic reviews that included RCCTs or CCTs in combination with non-experimental designs. There were no restrictions for any specific language, as recommended by the international criteria [18].

### 2.2. Search Strategy

We conducted the search for scientific articles on the following databases from their inception to March 2022: PubMed (Medline), PEDro, Web of Science, CINAHL, PsycINFO, and Google Scholar. The search strategy combined medical subject headings (MeSH) and non-MeSH terms, adding a Boolean operator (OR, NOT, and/or AND) to combine them (Appendix A). The search was conducted by two independent reviewers (C.V.R. and L.S.M.) using the same methodology. Differences that emerged during this phase were resolved by consensus. The reference sections of the original studies were screened manually, and the authors were contacted for further information if necessary.

### 2.3. Selection Criteria and Data Extraction

Initially, two independent reviewers (J.C.G. and L.S.M.) conducted a screening assessing the relevance of the systematic reviews (with and without meta-analyses) regarding the studies’ questions and objectives. The first screening was based on each study’s title information, abstract, and keywords. The full text was reviewed if there was no consensus or if the abstracts contained insufficient information. In the second phase of the screening, the full text was assessed if the studies met all of the inclusion criteria. Differences between the reviewers were resolved by a discussion and consensus process mediated by a third reviewer (F.C.M.). The data described in the Results section were extracted by means of a structured protocol that ensured that the most relevant information was obtained from each study.

### 2.4. Methodological Quality Assessment

Two independent reviewers (F.C.M. and L.S.M.) assessed the methodological quality of the systematic reviews (with or without meta-analyses), assessing each of the selected studies based on the Modified Quality Assessment Scale for Systematic Reviews (AMSTAR) developed by Barton et al. [19]—a scale shown to be a valid and reliable tool for assessing the methodological quality of systematic reviews. With a total of 13 items, each worth 2 points (with “yes” scoring 2, “in part” scoring 1, and “no” scoring 0), the maximum possible score is 26. A high-quality cutoff of 20 or more points was provided by the developers. The exclusion and keyword criteria were modified to better evaluate the selected systematic reviews in this study. In addition, we calculated the kappa coefficient (κ) and percentage (%) agreement scores to assess reliability prior to any consensus, and estimated the inter-rater reliability using κ: (1) κ > 0.7 indicates a high level of agreement between the reviewers, (2) κ of 0.5–0.7 indicates a moderate level of agreement, and (3) κ < 0.5 indicates a low level of agreement [20]. Disagreements on the final quality assessment score were resolved by consensus with a third independent reviewer (J.C.G.).

#### 2.4.1. Risk of Bias Assessment

We assessed the risk of bias with the Risk of Bias in Systematic Reviews tool (ROBIS), which consists of 3 phases: (1) relevance assessment (optional); (2) identification of concerns with the review process through 4 domains related to study eligibility criteria, identification and selection of studies, data collection and study appraisal, and synthesis and findings; and (3) judgment on the risk of bias. The ROBIS tool includes signaling questions to evaluate specific domains to help judge the systematic review’s risk of bias, which should be answered as “yes”, “probably yes”, “probably no”, “no”, or “no information”. The risk of bias is therefore judged as “low”, high”, or “unclear” [21]. Two independent reviewers (F.C.M. and L.S.M.) evaluated the risk of bias in the selected studies. Disagreements were resolved through consensus and mediation by a third reviewer (J.C.G.).

#### 2.4.2. Grading of Evidence

The Physical Activity Guidelines Advisory Committee (PAGAC) grading criteria were used to assess the grading of evidence. The criteria used to assess the quality of the evidence were as follows: (1) applicability of the study sample, exposures, and outcomes to the research question; (2) generalizability to the population of interest; (3) risk of bias/study limitations; (4) quantity and consistency of findings across studies; and (5) magnitude and precision of the effect(s). With these data, final evidence grades and conclusion statements for each research question were developed [22].

## 3. Results

### 3.1. Study Selection

**Figure 1:**
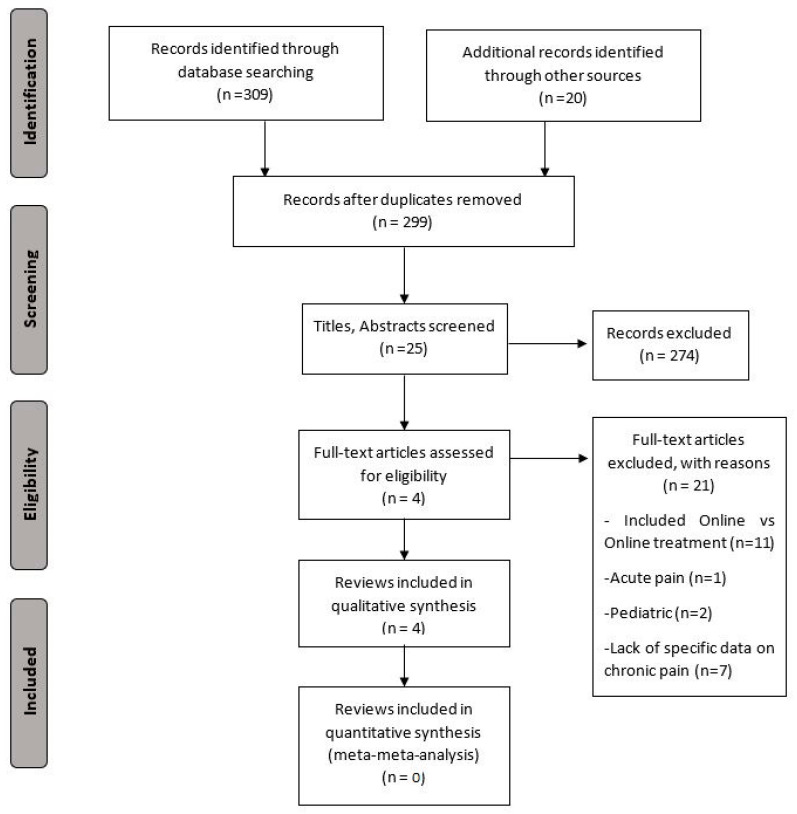
PRISMA flowchart of study selection.

The initial search revealed 309 records, and an additional 20 were retrieved manually from the references. Through the title and abstract screening and the full-text assessment, only four systematic reviews were eligible according to our criteria [6,13,14,15]. The study screening strategy is shown in the form of a flowchart (Figure 1). In total, 25 randomized controlled trials were included in the different systematic reviews. Only 16% (4/25) were used more than one time.

### 3.2. Characteristics of the Included Systematic Reviews

This umbrella review included a total of 4593 participants. Table 1 lists the characteristics of the systematic reviews included (i.e., study design, original studies included, demographic characteristics, interventions, variables, and results).

Two systematic reviews included chronic lower-back pain [6,13], and two included different types of chronic pain patients, such as fibromyalgia, chronic widespread pain, or headaches and migraine [14,15].

Interventions were mainly Internet-based; only one also included the use of mobile-based interventions [14]. They implemented different BMTs, such as ACT, CBT, education, self-management, exercise, exposure therapy, mindfulness, online feedback, or social support through an e-community. The clinical effects of these interventions were assessed in comparison to the effects of no or minimal intervention, or of an equivalent (or not) in-person intervention. Table 2 lists the details of the different interventions applied.

### 3.3. Results of the Methodological Quality

The scores ranged from 19 to 23 points out of a possible 26, with a mean score of 20.25 ± 3.12 points. Only one (25%) study scored above 20 points and was considered high-quality (Table 3). The items with the highest scores were those related to the number of databases (the acceptable number was considered to be more than three databases [19]) and the inclusion and exclusion criteria. The lowest-scoring item was the consideration of the level of evidence in the conclusion of the review. The inter-rater reliability of the methodological quality assessment was high (κ = 0.72).

### 3.4. Results of Risk of Bias

**Figure 2:**
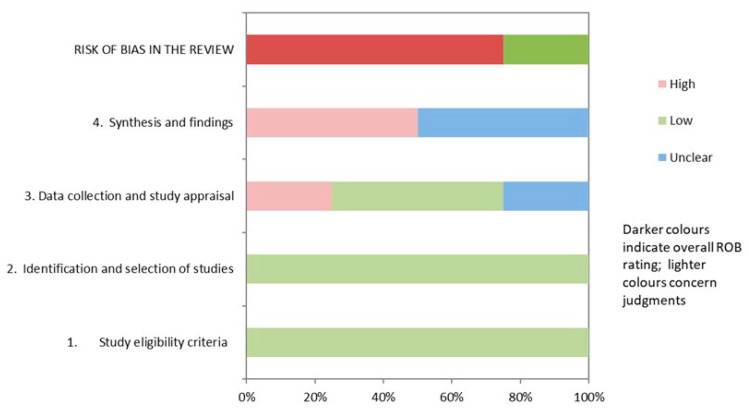
Graphical representation of the ROBIS results.

Table 4 and Figure 2 show the results of the risk of bias assessment using ROBIS. In total, 25% of studies had a low risk of bias. The domains related to the “study eligibility criteria” and the “identification and selection of studies” had the lowest risk of bias (100%). In contrast, the domain related to the “synthesis and findings” had the highest risk of bias (50%). The inter-rater reliability for the risk of bias assessment was high (κ = 0.78).

### 3.5. Grading of Evidence Results

Table 5 shows the findings regarding the quality of evidence for each outcome of the research question. The quality of evidence found for all outcome measures was limited.

### 3.6. Qualitative Synthesis

#### 3.6.1. Pain Intensity

Three reviews with and without meta-analyses assessed the effectiveness of t-BMT on pain intensity in patients with chronic lower-back pain in the short term [6,13,14]. Ariza-Mateos et al. [14] found that t-BMT improved pain intensity in women with chronic pain. Similar results were found by Du et al. [6], who obtained a small effect size in favor of t-BMT on pain intensity (SMD = −0.26; 95% CI −0.42 to −0.09). However, Darío et al. [13] found a non-significant effect in favor of t-BMT in patients with chronic lower-back pain (SMD = −0.05; 95% CI −0.10 to 0.00).

In addition, one review with a meta-analysis showed that *t*-BMT had no significant effect (SMD = −0.01; 95% CI: −0.74 to 0.72) on pain intensity in chronic lower-back pain in the medium term [13].

#### 3.6.2. Disability

Two reviews with meta-analyses assessed the effectiveness of t-BMT on disability in patients with chronic lower-back pain in the short term [6,13]. Both articles showed statistically significant differences in favor of t-BMT in patients with lower-back pain in the short term. First, Du et al. [6] showed a significant improvement in disability with a small effect size of t-BMT (SMD = −0.34; 95% CI −0.50 to −0.17). Second, Darío et al. [13] found a trivial effect size of t-BMT on disability in the short term (SMD = −0.04; 95% CI −0.07 to −0.02).

However, one review with a meta-analysis showed that t-BMT had no significant effect (SMD = 0.00; 95% CI: −0.06 to 0.07) on disability in chronic lower-back pain in the medium term [13].

#### 3.6.3. Psychological Symptoms

Two reviews with and without meta-analyses found that t-BMT was not superior to in-person treatment (CBT), but had positive effects compared to waiting lists on psychological distress in fibromyalgia (one primary study; *n* = 40; SMD = 2.647; SE = 0.429) [14,15]; however, both reviews included the same primary study. One review with a meta-analysis found that t-BMT had no positive effects in unspecific chronic pain compared to usual care (one primary study; *n* = 63; SMD = −0.051; SE = 0.249) [15]. Timepoints of the follow-up results were unclear.

The other meta-analysis, against usual care or waiting lists, showed a small-to-moderate effect size of t-BMT on depressive symptoms in patients with unspecific chronic pain (eight primary studies; *n* = 1418; SMD = 0.372; SE = 0.128) and fibromyalgia (four primary studies; *n* = 305; SMD = 0.679; SE = 0.259) but no statistically significant effect on migraine and headache (three primary studies; *n* = 320; SMD = 0.142; SE = 0.120) [15]. They also showed a small effect size on anxiety in patients with unspecific chronic pain (seven primary studies; *n* = 1355; SMD = 0.372; SE = 0.128), but no statistically significant effect on migraine and headache (two primary studies; *n* = 275; SMD = 0.422; SE = 0.301) or fibromyalgia (one primary study; *n* = 118; SMD = 0.046; SE = 0.183) [15]. Timepoints of the follow-up results were unclear.

One review without a meta-analysis found that t-BMT seems to be effective in improving pain-related fear of movement in patients with fibromyalgia compared to usual care (one primary study; *n* = 67, *p* = 0.001) [14]. Timepoints of the follow-up results were unclear.

#### 3.6.4. Disease Impact

One review without a meta-analysis found that t-BMT seems to be effective in improving disease impact in patients with fibromyalgia compared to usual care or waiting lists (five primary studies; *n* = 403; *p* = 0.0006 to *p* < 0.001) [14]. However, it does not seem to be superior to in-person BMT. Timepoints of the follow-up results were unclear.

#### 3.6.5. Physical Function

One review without a meta-analysis found that t-BMT does not seem to be an effective modality to improve the physical function of patients with chronic lower-back pain in the short (one primary study; *n* = 229) and medium term (one primary study; *n* = 334), compared to minimal intervention [13].

## 4. Discussion

### 4.1. Principal Results

The main aim of this umbrella review was to evaluate the effects of t-BMT on pain intensity, disability, psychological distress, depressive symptoms, anxiety symptoms, physical function, pain-related fear of movement, and disease impact in patients with chronic musculoskeletal pain. Our results showed that t-BMT was not effective in improving physical function in chronic lower-back pain compared with minimal intervention. However, t-BMT seemed to be effective in improving disability, disease impact, and pain-related fear of movement in fibromyalgia when compared with waiting lists or usual care. In addition, it seems that there was mixed evidence in favor of t-BMT regarding pain intensity, psychological distress, and anxiety and depressive symptoms. Regarding the comparison between telematic vs. in-person BMTs, we found no differences in psychological distress and disease impact in fibromyalgia. No other variables reported this comparison. Finally, t-BMT did not seem to be effective in improving anxiety and depressive symptoms in migraine and headache compared with waiting lists or usual care. However, it was effective in improving depressive symptoms and anxiety in an unspecific chronic pain population compared with waiting lists or usual care.

Due to the COVID-19 pandemic, there is growing scientific and societal interest in telerehabilitation. A recent systematic review suggested that telerehabilitation is a strategy comparable to conventional rehabilitation in patients with cardiac or neurological disorders [8]. However, our results do not support this benefit in the totality of variables studied in patients with chronic musculoskeletal pain. It is necessary to highlight the complex nature of rehabilitation for these patients. Chronic musculoskeletal pain depends on a multifactorial interaction of biological, psychological, and social factors [10,23]. It remains challenging to develop conventional treatments that are beneficial for these patients, because many of the current treatments are ineffective. Because telerehabilitation is based on these treatments, it is therefore possible that the results are similar to those of conventional treatments. In addition, previous research has found that telerehabilitation may result in lower patient adherence or confidence, which may decrease the treatment’s effectiveness [24]. Furthermore, difficulties in assessing some outcomes (e.g., physical function) during telerehabilitation might negatively impact the validity and reliability of the results. However, the results of telerehabilitation on psychological symptoms and disease impact reveal the therapeutic potential of these interventions in the management of chronic musculoskeletal pain. In this direction, future trials should evaluate the impact of the time of intervention, as well as the resources used for the procedure, since it seems that longer interventions using face-to-face telerehabilitation systems accompanied by social support may be more effective in this population [25].

### 4.2. Gap in the Literature

Most of the systematic reviews included were rated as having a high risk of bias. Future reviews should improve on this to increase our confidence in their results. The effect of a technique may be overestimated when we compare it with no intervention [26]. Future systematic reviews should try to avoid pooling studies that compare t-BMT with minimal intervention and studies that compare it with another in-person active intervention in the same statistical analysis, because the effect(s) may be different. We excluded a large proportion of systematic reviews because they did not include only studies evaluating telerehabilitation versus in-person treatment, but compared one type of telerehabilitation versus another. Future reviews should also differentiate studies in different sub-analyses. We found that t-BMT is not effective in patients with chronic lower-back pain; however, there is a necessity to evaluate its effects on other populations to increase its external validity. Future systematic reviews should evaluate the effects of different electronic formats (e.g., synchronous/non-synchronous, on mobile, laptop, tablet, etc.) in different sub-analyses to determine whether all of the different formats are suitable for implementing behavior modification techniques.

### 4.3. Limitations and Strengths

We found a limited strength of evidence for our results; there is a need to follow to investigate the effects of t-BMT on patients with chronic musculoskeletal pain. The meta-analyses we included considered nonspecifically different types of interventions (CBT, ACT, exposure behavioral therapy, or mindfulness) and different electronic formats (internet-based or mobile-based), so our estimated effect might be different from the true therapeutic effect of t-BMT. In addition, another issue identified is the high risk of bias in some of the studies. With only four included, this is a major limitation. This was found to be due to minor problems in some areas of interest, such as the synthesis of the findings, or the data collection and evaluation of the studies. To improve these aspects for the future, the authors should describe methods of data collection, what data were extracted from studies or collected through other means, how the risk of bias was assessed (e.g., the number of reviewers involved), and the tool used to assess the risk of bias or describe the synthesis methods. In addition, we were not able to assess publication bias through funnel and Doi plots, because only two meta-analyses were compared. This should be taken into consideration as a limitation. Finally, it is important to stress that the Google Scholar database is very limited in terms of Boolean search operators, so it is almost impossible to replicate a search in this database, and this must be made public.

## 5. Conclusions

The results obtained show that t-BMT was effective in improving disability, pain-related fear of movement, and disease impact compared to minimal intervention, no intervention, or usual care, but not in improving physical function, with a limited quality of evidence. Mixed evidence was found regarding pain intensity, psychological distress, and anxiety and depressive symptoms—also with a limited quality of evidence. Finally, t-BMT was as effective as in-person BMT for the management of psychological distress and disease impact.

## Figures and Tables

**Table 1:** Characteristics of the reviews included in the umbrella review.

Study	Number of Studies, Study Design (Sample)	Patient Characteristics	Intervention (Type of Technology) and Control Group	Outcomes		
				Nº of Studies Includes in Meta-Analysis (Participants)	Scales of Measurement	Results
Ariza-Mateos et al. [14]	7 RCTs ^a^ (*n* = 562)	*Fibromyalgia, chronic widespread pain, or chronic primary headaches* 100%F ^b^ Age: 32.5 to 53.5 yrs ^c^	*Intervention* ACT ^d^, CBT ^e^, self-management, exercise, exposure therapy, and mindfulness (Internet-based or mobile-based) *Control* Control intervention or in-person treatment	*Pain intensity*		
				N/A ^f^	VAS ^g^, McGill Pain Questionnaire, DASS-21 ^h^	t-BMT ^i^ seemed to be effective to improve pain intensity in women with chronic pain.
				*Pain-related fear of movement*		
				N/A	Tampa scale of kinesiophobia	t-BMT seemed to be effective to improve pain/fear avoidance in women with chronic pain
				*Disease impact*		
				N/A	Fibromyalgia impact questionnaire; fibromyalgia impact questionnaire—revised	t-BMT seemed to be effective to improve the impact of fibromyalgia on women with chronic pain
				*Psychological distress*		
				N/A	HADS ^j^, GAD-7 ^k^	t-BMT seemed to be effective to improve anxiety and depression in women with chronic pain.
White et al. [15]	15 RCTs	*Chronic pain, fibromyalgia, and chronic headache/ migraine* 18 to 100%F	*Intervention* Psychology-based interventions (Internet-based) *Control* Usual care or waiting list	*Depressive symptoms*		
				Chronic pain: 8 RCTs Fibromyalgia: 4 RCTsHeadache/ migraine: 3 RCTs	HADS, PHQ ^l^, CES-D ^m^, BDI ^n^	t-BMT showed a trivial effect size on migraine/headache (Hedge’s g = 0.142; SE ^o^ = 0.120), small effect size in chronic pain patients (Hedge’s g = 0.372; SE = 0.128), and a moderate effect size on fibromyalgia (Hedge’s g = 0.679; SE = 0.259) and depressive symptoms.
				*Anxiety*		
				Chronic pain: 7 RCTs Headache/migraine: 2 RCTs	HADS, GAD-7, DASS ^p^	t-BMT showed a small effect size on patients with chronic pain (Hedge’s g = 0.236; SE = 0.090) and on patients with migraine/ headache (Hedge’s g = 0.422; SE = 0.301) or anxiety.
Darío et al. [13]	4 RCTs (*n* = 1342)	*Chronic LBP ^q^* 13 to 68%F Age: 46 to 51 yrs	*Intervention* Behavioral intervention, CBT, exercise feedback, e-community (Internet-based) *Control* Usual care, e-mail, equivalent in-person intervention, magazine subscription	*Pain intensity*		
				Short term: 4 RCTs Medium term: 2 RCTs	N/R ^r^	Non-significant effect of t-BMT on pain intensity in the short term (SMD ^s^ = −0.05; 95%CI ^t^ −0.10,0.00) and medium term (SMD = −0.01; 95%CI −0.74, 0.72).
				*Disability*		
				Short term: 4 RCTs Medium term: 2 RCTs	N/R	Trivial effect size of t-BMT on disability in the short term (SMD = −0.04; 95%CI −0.07, −0.02), but non-significant in the medium term (SMD = 0.00; 95%CI −0.06, 0.07).
				*Physical function*		
				N/A	N/R	t-BMT did not seem to be superior to the control group in improving physical function.
Du et al. [6]	3 RCTs (*n* = 590)	*Chronic LBP* 60.6 to 83.0%F Age: 21.0 to 44.6 yrs	*Intervention* Self-management (Internet-based) *Control* Waiting list or usual care	*Pain intensity*		
				3 RCTs	N/R	Small effect size of t-BMT on pain intensity (SMD = −0.26; 95% CI −0.42, −0.09).
				*Disability*		
				3 RCTs	N/R	Small effect size of t-BMT on disability (SMD = −0.34; 95% CI −0.50, −0.17).

^a^ RCT: randomized controlled trial. ^b^ % F: proportion of female. ^c^ yrs: years. ^d^ ACT: acceptance and commitment therapy. ^e^ CBT: cognitive behavioral therapy. ^f^ N/A: not applicable. ^g^ VAS: Visual Analogue Scale. ^h^ DASS-21: 21-Item Depression, Anxiety, and Stress Scale. ^i^ t-BMT: telerehabilitation based on behavioral modification techniques ^j^ HADS: Hospital Anxiety and Depression Scale. ^k^ GAD-7: 7-item Generalized Anxiety Disorder Scale. ^l^ PHQ: Patient Health Questionnaire. ^m^ CES-D: Center for Epidemiological Studies–Depression. ^n^ BDI: Beck Depression Inventory. ^o^ SE: standard error. ^p^ DASS: Depression, Anxiety, and Stress Scale. ^q^ LBP: lower-back pain. ^r^ N/R: not reported. ^s^ SMD: standardized mean difference. ^t^ 95%CI: 95% confidence interval.

**Table 2:** Intervention analysis.

Author, Year	Intervention Group	Electronic Format	Control Group	Frequency	Intervention Duration
Ariza-Mateos et al. [14]	- *45 min exercise* (aerobic, strength, and stretching training) *and education about the disease;* - *120 min CBT ^a^* (education about the disease; relaxation and breathing training); - *Online lessons* (information, cognitive therapy, and pain management); - *Exposure therapy* (education, case examples, mindfulness exercises, and treatment based on goals); - *Acceptance and commitment therapy* (education, values, cognitive defusion, mindfulness, willingness and committed action, feedback, and daily diaries); - *Mindfulness-based stress reduction.*	Internet-based and smartphone-delivered	Control group or in-person intervention	2 to 10 times per week	8 to 10 weeks
White et al. [15]	- *CBT* (Information, feedback, goal setting, conversation with the therapist, homework, answering questions, patient discussion of experiences, questionnaire to target information, relaxation, watching videos/demonstrations); - *Education;* *- Self-management* (feedback, goal setting, watching videos, online workshops, participation in a discussion board, questionnaire to target information); - Questionnaires targeting information or feedback, reminders, listening to real or simulated patients’ experiences, goal setting, skills, motivational messages, feedback graphs, homework or answering questions, watching videos/demonstrations, conversation with the therapist; - *Mp3 ^b^ audio recordings;* *- Relaxation;* *- Mindfulness;* *- Writing exercises.*	Internet-based	Usual care or waiting list	N/R ^c^	4 weeks to 6 months
Dario et al. [13]	- *E-mail discussion group, videotape about behavior changes, and book;* *- Self-management and CBT*; *- Pedometer + E-community, web-based walking program, and goal setting*; *- E-community, self-monitoring, and goals*.	Internet-based	Subscription to a non-health magazine, LBP ^d^ guide, pedometer only, and usual care	N/R	4 weeks to 12 months
Du et al. [6]	- *Self-help treatment* (education, cognitive skill, behavioral rehearsal, generalization, and maintenance); - *CBT* (cognitive therapy, behavioral activation, ACT ^e^, and mindfulness-based stress reduction); - *Self-tailored cognitive-behavioral approach* (prevention of pain behaviors).	Internet-based	Waiting list	N/R	3 to 8 weeks

^a^ CBT: cognitive behavioral therapy. ^b^ Mp3: MPEG-1/2 Audio Layer III. ^c^ N/R: not reported. ^d^ LBP: lower-back pain. ^e^ ACT: acceptance and commitment therapy.

**Table 3:** Quality assessment scores.

Study	1	2	3	4	5	6	7	8	9	10	11	12	13	Score
Ariza-Mateos et al. [14]	2	2	0	2	0	1	2	0	2	2	2	2	0	19
White et al. [15]	2	2	1	2	0	2	2	1	2	2	2	2	1	19
Dario et al. [13]	2	2	2	2	0	2	2	1	2	2	2	1	0	20
Du et al. [6]	2	2	2	2	1	2	2	2	2	2	2	2	0	23

1: Explicitly described to allow replication (i.e., 100% confident that you could replicate it) if explained, but cannot be 100% confident of replication. 2: Adequate number and range of databases. 3: Alternative searches. 4: Adequate range of key words. 5: Non–English-language papers included in the search. 6: Inclusion criteria explicitly described to allow replication. 7: Excludes reviews that do not adequately address inclusion. 8: Two independent reviewers assessing selection bias. 9: Quality assessment explicitly described to allow replication. 10: Meta-analysis conducted on only homogenous data, or limitations to homogeneity discussed. 11: CIs/effect sizes reported where possible. 12: Conclusions supported by meta-analysis or other data analysis findings (effect sizes, CI, etc.) in the review. 13: Conclusions address levels of evidence for each intervention/comparison. Scoring: 2 = yes; 1 = in part; 0 = no.

**Table 4:** Risk of bias assessment in systematic reviews through the ROBIS scale.

Study	Phase 2				Phase 3
	1. Study Eligibility Criteria	2. Identification and Selection of Studies	3. Data Collection and Study Appraisal	4. Synthesis and Findings	Risk of Bias in the Review
Ariza-Mateos et al. [14]	☺ ^a^	☺	☹ ^b^	? ^c^	☹
White et al. [15]	☺	☺	?	☹	☹
Dario et al. [13]	☺	☺	☺	☹	☹
Du et al. [6]	☺	☺	☺	?	☺

^a^☺: low risk. ^b^☹: = high risk. ^c^?: unclear risk.

**Table 5:** Summary of findings and quality of evidence (PAGAC).

2018 Physical Activity Guidelines Advisory Committee Grading Criteria						Grade
Systematic Review Research Questions	Applicability	Generalizability	Risk of Bias or Study Limitations	Quantity and Consistency	Magnitude and Precisionof Effect	
*Pain Intensity*	Strong	Limited	Limited	Limited	Not assignable	Limited
*Disability*	Strong	Limited	Limited	Limited	Not assignable	Limited
*Pain-related fear of movement*	Moderate	Limited	Limited	Limited	Not assignable	Limited
*Disease impact*	Moderate	Limited	Limited	Limited	Not assignable	Limited
*Psychological distress*	Moderate	Limited	Limited	Limited	Not assignable	Limited
*Depressive symptoms*	Strong	Limited	Limited	Limited	Not assignable	Limited
*Anxiety symptoms*	Strong	Limited	Limited	Limited	Not assignable	Limited
*Physical function*	Strong	Limited	Limited	Limited	Not assignable	Limited

## Appendix A. PubMed Systematic Search Strategy

((“Web”) OR (“Cell phone” [MesH]) OR (“eTherapy”) OR (“Internet”) OR (“Online”) OR (“Telerehabilitation”) OR (“Internet-Based Intervention” [MesH]) OR (“Telerehabilitation” [MesH]) OR (Telemedicine [MesH])) AND ((“Chronic Pain”) OR (“Chronic Pain” [Mesh])) AND ((((systematic review [ti] OR systematic literature review [ti] OR systematic scoping review [ti] OR systematic narrative review [ti] OR systematic qualitative review [ti] OR systematic evidence review [ti] OR systematic quantitative review [ti] OR systematic meta-review [ti] OR systematic critical review [ti] OR systematic mixed studies review [ti] OR systematic mapping review [ti] OR systematic cochrane review [ti] OR systematic search and review [ti] OR systematic integrative review [ti]) NOT comment [pt] NOT (protocol [ti] OR protocols [ti])) NOT MEDLINE [subset]) OR (Cochrane Database Syst Rev [ta] AND review [pt]) OR systematic review [pt]).
